# The Rotavirus Vaccine Story: From Discovery to the Eventual Control of Rotavirus Disease

**DOI:** 10.1093/infdis/jiaa598

**Published:** 2021-09-30

**Authors:** Roger I Glass, Jacqueline E Tate, Baoming Jiang, Umesh Parashar

**Affiliations:** 1 Viral Gastroenteritis Branch, Centers for Disease Control and Prevention, Atlanta, Georgia, USA; 2 Fogarty International Center, National Institutes of Health, Bethesda, Maryland, USA

**Keywords:** rotavirus, vaccine, child survival, childhood diarrhea

## Abstract

Worldwide, rotavirus is the leading pathogen causing severe diarrhea in children and a major cause of under 5 years mortality. In 1998, the first rotavirus vaccine, RotaShield, was licensed in the United States but a rare adverse event, intussusception, led to its withdrawal. Seven years passed before the next generation of vaccines became available, Rotarix (GSK) and Rotateq (Merck), and 11 years later, 2 additional vaccines from India, Rotavac (Bharat) and Rotasiil (Serum Institute), were recommended by World Health Organization for all children. Today, these vaccines are used in more than 100 countries and have contributed to marked decreases in hospitalizations and deaths from diarrhea. However, these live oral vaccines are less effective in low-income countries with high under 5 years mortality for reasons that are not understood. Efforts to develop new vaccines that avoid the oral route are in progress and will likely be needed to ultimately control rotavirus disease.

One of the public health miracles of the 20th century has been the prolongation of our life expectancy from about 40 years at the turn of the 20th century to nearly 70 years by the beginning of the 21st century [1]. This improvement has been attributed, in part, to the 80% decline in under 5 years mortality (U5M) from 1900, when about 1 in 5 children died before their fifth birthday, until today when 24 of 25 (96%) survive. Survival is still largely linked to economic status, where a child is born, health care provided, education of the mother, income level of the country, and war and civil unrest. These improvements in life expectancy have not been shared equally. Even today in some communities in the world’s poorest countries and communities, these same statistics remain. Today, diarrhea remains one of the most common illnesses of children younger than 5 years and in low-income settings it remains one of the top 2 or 3 causes of death [2].

In the 1970s, several groups interested in child survival made crude estimates of the global burden of diarrhea in children younger than 5 years [3–6]. While these estimates ranged widely from 3 to 12 million deaths per year, they did focus attention on the critical role that diarrhea played as a determinant of child survival. The medical and public health communities were challenged to improve child survival through research by “taking science to where the problems were” [7]. A group of longitudinal studies documented that children in Bangladesh [8], Guatemala [5], and Peru [9] had from 4 to 8 episodes of diarrhea per child per year during their first 5 years of life, 20–40 episodes in total, and these episodes ranged in duration from 2 days to more than 10 days [5]. A child would literally spend several months of their first 5 years living with diarrhea and these episodes were also associated with faltering growth. These striking data focused the attention of pediatricians and public health specialists on the prospect that the prevention and treatment of diarrheal illnesses needed to be a key target to improve child survival.

To bring science to the problem, researchers had to identify the causes of diarrhea by both their etiologies and their distinct modes of transmission. At the time, a pathogen could only be identified for fewer than 15% of these episodes—a few bacteria (Vibrio cholerae, *Shigella*, *Salmonella*), several parasites (*Entamoeba histolytica* and *Giardia lamblia*), and some environmental toxins. Cases left undiagnosed were considered idiopathic, and often attributed to conditions such as malnutrition or the introduction of weanling foods that might alter the physiologic function of these children. Because these diseases and deaths were more common in low-income countries— and some like cholera and amebiasis were known to be transmitted by fecally contaminated water, while others like *Salmonella* were often spread by contaminated food—interventions to improve hygiene, and the quality of food and water were considered the most important control measures.

## FROM DISCOVERY TO THE FIRST LICENSED ROTAVIRUS VACCINE

A key breakthrough occurred in 1972 when Albert Kapikian used immune electron microscopy (EM) to discover the Norwalk agent, the first virus identified to cause outbreaks of acute diarrhea in children and adults alike [10]. One year later, Ruth Bishop identified a wheel-shaped virus by EM in biopsy tissue from the duodenum of children with acute diarrhea that was later named rotavirus [11]. Over the next decade, more than a dozen different pathogens—bacteria, parasites, and viruses, and toxins—were discovered to cause acute diarrhea. For each of these agents, research focused on developing the best diagnostic tool, identifying the mode of transmission and the reservoir of infection, and determining the best strategies to prevent disease.

### Early Epidemiologic Studies

Establishing the epidemiology and burden of disease for rotavirus became an early priority. Studies in Bangladesh examined the importance of rotavirus in 2 settings, the community and the hospital. A longitudinal study following a birth cohort of Bangladeshi children found that these children reported 15–25 episodes of diarrhea during their first 5 years of life, but rotavirus was rarely detected more than once, representing only 3%–5% of the total number of episodes [8]. However, among children hospitalized for diarrhea in the same area, 20% were diagnosed with rotavirus, indicating that rotavirus was the pathogen most likely to cause severe disease. The availability of a simple enzyme immunoassay with both high sensitivity and specificity and the high concentration of virus, >10^10^ per gram of stool [10–12], then permitted laboratories around the world to regularly diagnose rotavirus from children admitted for diarrhea. Consequently, sentinel surveillance for rotavirus among children hospitalized for diarrhea was begun, is ongoing today in more than 60 countries, and has become a key measure to assess the burden of rotavirus disease and monitor the impact of vaccination programs. Worldwide, roughly 36% of these children younger than 5 years have rotavirus detected as their pathogen (range, 24%–50%) [12]. Infection is nearly universal and most children develop antibodies to rotavirus by the age of 5 years [13]. Furthermore, unlike cholera, shigella, and amebiasis, which occur primarily among children in low-income settings, all children worldwide are infected with rotavirus early in life, an observation that has led rotavirus to be branded as a “democratic virus” affecting rich and poor, and not sparing any geographic or social group. In the absence of a known mode of transmission, the winter seasonality suggested that respiratory spread might be involved.

In Mexico, a study of the natural history of rotavirus disease by Velazquez and Ruiz-Palacios documented that once children were infected with rotavirus, they were protected against severe disease upon reinfection, evidence that natural immunity was protective, thereby laying the groundwork for the vaccine. In 1986, the Institute of Medicine convened a group of experts to conduct a Delphi exercise to prioritize infectious diseases suitable for vaccine development [14]. The group reviewed the epidemiology of rotavirus and diarrheal deaths in many countries and estimated that worldwide, 873 000 (23%) of the 3.8 million diarrheal deaths per year in children were attributable to rotavirus. They concluded that the development of a rotavirus vaccine should receive its highest priority.

### Laboratory Studies Relevant to Vaccine Development

Between the discovery of rotavirus in 1973 and 1990, basic research cleared the path for vaccine development. A critical advance was discovering how to grow the virus in tissue culture, essential for determining serotypes by virus neutralization with convalescent sera and for the preparation of live oral vaccines [15]. Unravelling the molecular structure and gene coding assignments helped understand how in mixed infections, viral segments can reassort to form new strains, a process later used to develop more effective vaccines. Rotaviruses have been found in many mammalian species and birds, but because these strains or their genes can rarely be found in human strains, it seems unlikely that we will ever be able to truly eradicate rotavirus disease in humans.

**Figure 1.**
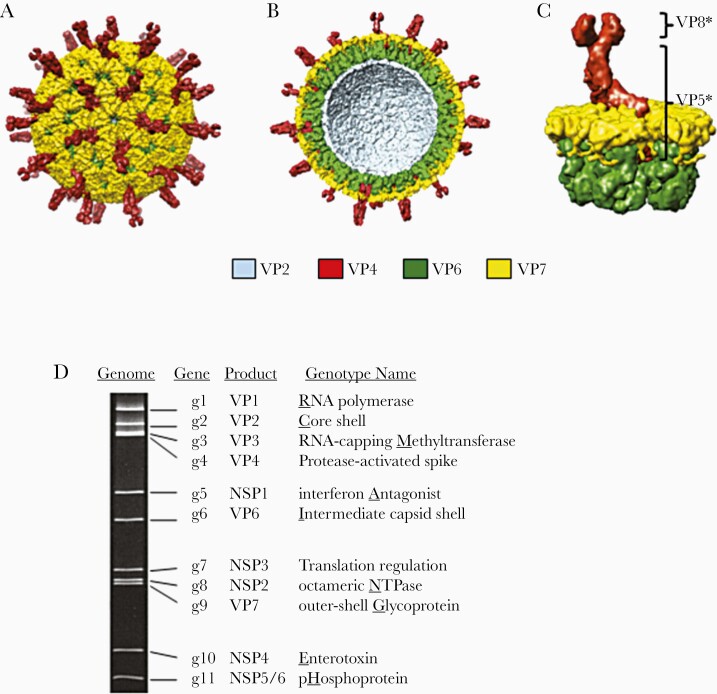
Rotavirus capsid structure and dsRNA genome. *A*, Intact triple-layered virion with VP4 spikes projecting from the VP7 outer capsid shell. *B*, Cut-away of the virion revealing the 3 protein layers of the virion: VP2, VP4, and VP7. *C*, A VP6 hexamer, VP7 hexamer, and embedded VP4 spike with the VP8* and VP5* regions identified. *D*, Eleven double-stranded RNA segments of the rotavirus genome resolved by gel electrophoresis. Segments are labeled as g1–g11 (g = gene) and their protein products are listed. Associated functions or properties of the products are given (genotype name). The underlined letter identifies the segment in the gene constellation acronym: for common assignment of serotypes (genotypes), only the Gx and Px are used (eg, G1,P8). Reprinted from Patton with permission [16].

The genome of rotavirus is composed of 11 segments of dsRNA easily visible by gel electrophoresis (Figure 1). Each segment encodes a single protein, except VP4 which is cleaved in 2, VP5 that is anchored to the intermediate capsid shell (VP6), and VP8, the hemagluttinin spike that attaches to the epithelial cells in the gut. These 11 segments are enclosed is a triple-layered shell with VP2 encoding the core shell, VP6 the intermediate capsid, and VP7 the outer capsid that is decorated with the VP4 spikes protruding from its surface. Classification of strains was initially based upon the 2 neutralization proteins, VP7, a glycoprotein or G-protein, and VP4, the protease-cleaved protein or P-protein with the most abundant strain being characterized by neutralization serotypes initially (eg, serotype 1 (G1,P8)) and subsequently by genotype indicated with brackets (eg, G1, P[8]). Viral segments can be reassorted in the laboratory and this has allowed reassortant vaccine strains to be constructed using rotavirus strains from animals, which are naturally attenuated for humans, with segments inserted that represent the major G- and P- serotypes found in humans [17–20]. Despite the opportunities for reassortment, the diversity of strains is relatively limited with clusters of gene segments that stick together and variants from animal rotaviruses that only occasionally find their way into strains infecting humans. Overall, there are only 5 generally common strains worldwide defined by their G- and P-type, but there can be a much greater variety, especially among children living in low-income settings.

### Early Vaccine Development

By 1983, only 10 years after the discovery of the virus, the first rotavirus vaccine entered clinical testing [21]. The vaccine, RIT 4237, derived from a bovine strain of rotavirus was tested in only 178 infants and proved to be safe, immunogenic, and provided significant protection against severe disease [21]. This groundbreaking trial identified 4 enduring principles for the development of live oral rotavirus vaccines: the vaccine was effective in children; efficacy was greatest against the most severe disease versus milder disease; the immune response measured by immunoglobulin A (IgA) antibodies to rotavirus did not predict vaccine efficacy (ie, it was not a correlate of protection); and like Jenner observed with smallpox, a bovine strain (cowpox) could protect humans against disease. Because the bovine strain contained neither the VP7 or VP4 segments of human strains, the proteins responsible for neutralizing activity against human rotaviruses, immunity to rotavirus must therefore reside on multiple gene segments and with many different epitopes. Unfortunately, RIT 4237 and 2 other early monovalent vaccine candidates based upon single animal strains, WC3, a bovine strain, and Rhesus rotavirus (RRV), a monkey strain, demonstrated variable efficacy in children and were discarded from further development. However, investigators recognized that because these animal strains did not cause disease in humans, they were naturally attenuated and might be rendered more protective if they were reassorted to carry the G- and P-gene segments, VP7 and VP4, commonly found in strains from humans that were responsible for virus neutralization [22]. Other live oral rotavirus vaccines were developed either from strains pathogenic in humans that were attenuated by serial passage in cell culture or naturally attenuated when isolated from infected newborns who did not develop diarrhea.

The first live oral vaccine to be licensed was a tetravalent Rhesus reassortant vaccine (RRV-TV) composed of a rotavirus strain obtained from a rhesus monkey, RRV, that was combined with 3 reassortant strains each containing 10 segments from the original RRV strain plus a single VP7 outer capsid gene segment encoding the glycoprotein from 1 of the 3 common rotavirus strains in humans, serotypes G1, G2, and G4, while RRV carried the G3 VP7 gene [23]. RRV-TV, licensed as RotaShield (Wyeth Lederle), demonstrated significant protection against rotavirus diarrhea of any severity (range, 49%–68%) and greater protection against severe rotavirus diarrhea (range, 61%–91%) [24–28]. Following its licensure in 1998, RotaShield was immediately recommended for use in all American children as 3 oral doses given at 2, 4, and 6 months of age [29].

Within 9 months, 600 000 children had received the vaccine and it seemed like the worldwide distribution of a rotavirus vaccine would soon follow. Then, intussusception (IS), a troubling adverse event, was detected that altered vaccine history [30]. Postlicensure data identified a small increase in the number of cases of IS in the week following administration of the first dose, an excess of approximately 1 IS case per 10 000 vaccinated infants [31]. The Centers for Disease Control and Prevention (CDC) halted the use of the vaccine during the investigation and the company withdrew the product from the market several months later [32]. Small studies to test the plausibility of the association have found that rotavirus infection is associated with increased distal ileum wall thickness and lymphadenopathy during the acute illness period [33]. However, epidemiologic studies found no seasonality of intussusception that matched the distinct winter seasonality of rotavirus in temperate climates [34]. Consequently, the mechanism for this association has never been determined but the fact that risk was greatest in the first week following the first dose of the vaccine suggested that it was related to intestinal replication of the live vaccine virus.

## CURRENT LICENSED ROTAVIRUS VACCINES

The abrupt and unanticipated withdrawal of RotaShield was a major setback to global rotavirus prevention efforts. Furthermore, given the lack of a clear biologic mechanism for the association of RotaShield with IS, 2 manufacturers of other candidate live oral rotavirus vaccines faced the daunting prospect of conducting large and expensive clinical trials to demonstrate whether their candidates were safer. In 2006, 2 new live oral rotavirus vaccines were licensed in the United States—a monovalent 2-dose vaccine based on an attenuated strain of human rotavirus G1P[8] (Rotarix; GlaxoSmithKline) and a 3-dose pentavalent bovine rotavirus based vaccine containing 5 single-gene reassortant strains bearing capsid proteins for human serotypes G1, G2, G3, G4, and P[8] (RotaTeq; Merck) (Table 1) [35, 36]. The trials were massive, more than 60 000 infants, and sized to ensure the safety of each vaccine against IS. Efficacy of 85%–95% was documented against severe disease or hospitalization but both were less effective against milder illness. Following licensure and introduction of RotaTeq in the United States in 2006, the World Health Organization (WHO) recommended rotavirus vaccine for routine use in children in high- and middle-income countries where efficacy had been demonstrated [37]. Many countries in these regions, beginning with the United States, implemented programs for the routine immunization of all children against rotavirus.

**Table 1.** Live Oral Rotavirus Vaccines

Vaccine, Manufacturer	Year	Principle Strains	Formulation, Volume, Stability, Presentation	No. of Doses	Comments
World Health Organization prequalified					
Rotarix, GSK	2009	Monovalent Attenuated G1P[8]	Liquid 1.5 mL 2–8°C, 24 mo 1 dose tube/applicator	2	Most widely used
Rotateq, Merck	2008	Pentavalent human- bovine reassortants G1, G2, G3, G4, P[8]	Liquid 2.0 mL 2–8°C, 24 mo 1 dose tube	3	No longer supported by GAVI-2019
Rotavac, Bharat	2018	Monovalent Neonatal G9P[11]	Frozen 0.5 mL −20°C, 60 mo 5/10 dose vials	3	Most widely used in India
RotaSiil, Serum Institute of India	2018	Pentavalent human-bovine reassortants G1, G2, G3, G4, G9	Lyophil + buffer 2.5 mL 2–8°C, 30 mo 1 or 2 dose sets	3	Lyophil is thermostabile
Nationally licensed					
Lanzou, Lamb Lanzhou, IBP China	2000	Monovalent Lamb G10P[12]	Liquid + buffer 2.5 mL 2–8°C	4	Dosing, 1 dose/y for 3 y between 2 and 35 mo Polyvalent reassortant vaccine in development
Rotavin, Polyvac Vietnam	2008	Monovalent Attenuated G1P[9]	Liquid 2.5 mL	3	
In development					
RV3, Biopharma Indonesia Australia	…	Monovalent Neonatal G3P[6]		3	In phase 3 trials Possible neonatal dose

The WHO also suggested that both vaccines be tested in low-income settings before they could issue their recommendation for use worldwide. They reasoned that other live oral vaccines against polio, typhoid, and cholera had performed less favorably in low-income settings. Each company conducted additional clinical trials to secure WHO’s global recommendation. In Africa and Asia, these studies demonstrated distinctly lower efficacy (50%–64%) compared with that seen in more affluent settings [38, 39, 40]. Several factors were hypothesized to be responsible—neutralization of the vaccines titer due to rotavirus antibodies from the placenta, interference from simultaneous oral polio vaccine immunization, other enteric pathogens or the microbiome, or factors that might alter the infants’ ability to mount a protective immune response such as malnutrition or other infections [41]. However, given the public health benefits from a moderately effective rotavirus vaccine against a disease that killed so many children in low-income settings, the WHO in 2009 issued a recommendation for rotavirus to be included in the immunization programs for all children worldwide [42].

The disappearance of RotaShield also encouraged several emerging manufacturers to develop their own rotavirus vaccines with an eye to markets in low- and middle-income countries where 95% of the fatal cases occurred. In 2018, 2 Indian-made rotavirus vaccines—Rotavac (Bharat Biotech) and Rotasiil (Serum Institute)—were prequalified by the WHO, allowing their procurement by the United Nations Children's Fund (UNICEF) and the Global Alliance for Vaccines and Immunization (GAVI) and use in low-income countries [43]. Rotavac is a monovalent vaccine based on a naturally attenuated G9P[11] neonatal human rotavirus strain that was tested in infants in India and demonstrated an efficacy against severe rotavirus gastroenteritis of 56% (95% confidence interval, 37%–70%) for the first 2 years of life [44]. Rotasiil is a pentavalent bovine rotavirus reassortant vaccine based upon a different rotavirus strain than in RotaTeq (UK vs WC3 strains, respectively) that contains 5 single-gene reassortants with VP7 capsid proteins for human serotypes G1, G2, G3, G4, and G9. In clinical trials in India and Niger, Rotasiil demonstrated an efficacy of 36% (95% confidence intervals (CI), 12%–49%) and 67% (95% CI 50%–78%), respectively, against severe rotavirus gastroenteritis [45, 46]. While an increased intussusception risk was not seen in clinical trials of either Rotavac or Rotasiil, these trials enrolled <10 000 infants each and were not adequately powered to examine a low-level risk.

An important and unique feature in the development of Rotavac was the creative financial agreement between the Government of India, the Bill and Melinda Gates Foundation, and PATH with the manufacturer, Bharat Biotech, to ensure the availability of the vaccine at an affordable price for India and low-income countries [47]. The manufacturer received major funding to conduct the expensive phase 3 trial and some of the development costs in return for their commitment to provide the vaccine to India and GAVI in large volume and at low cost, tagged at approximately $1 per dose. Ultimately, both Rotavac and Rotasill are being provided to the Government of India and to GAVI at the lowest prices (<$1.00 per dose) and have entered into the global markets of low- and middle-income countries.

Other rotavirus vaccines have been nationally licensed for domestic use [48]. Rotavin-M1, a monovalent vaccine based on an attenuated G1P[8] human rotavirus strain, is in the private market in Vietnam [49]. In a prelicensure trial, 73% of vaccinees seroconverted and further effectiveness trials are being evaluated in a pilot studies in 2 Vietnamese provinces [49]. China has licensed the Lanzhou lamb rotavirus vaccine (LLR) vaccine, which is based on a monovalent lamb rotavirus strain (G10P[12]) [50]. Since its licensure in 2000, more than 60 million doses of LLR have been distributed in the local private market in China. Although no efficacy data from prelicensure trials are available, LLR has shown effectiveness of 35%–77% in several case-control evaluations conducted post licensure [51–53]. A trivalent formulation of LLR using reassortant technology is in development [48].

## GLOBAL IMPLEMENTATION AND IMPACT OF ROTAVIRUS VACCINES

**Figure 2.**
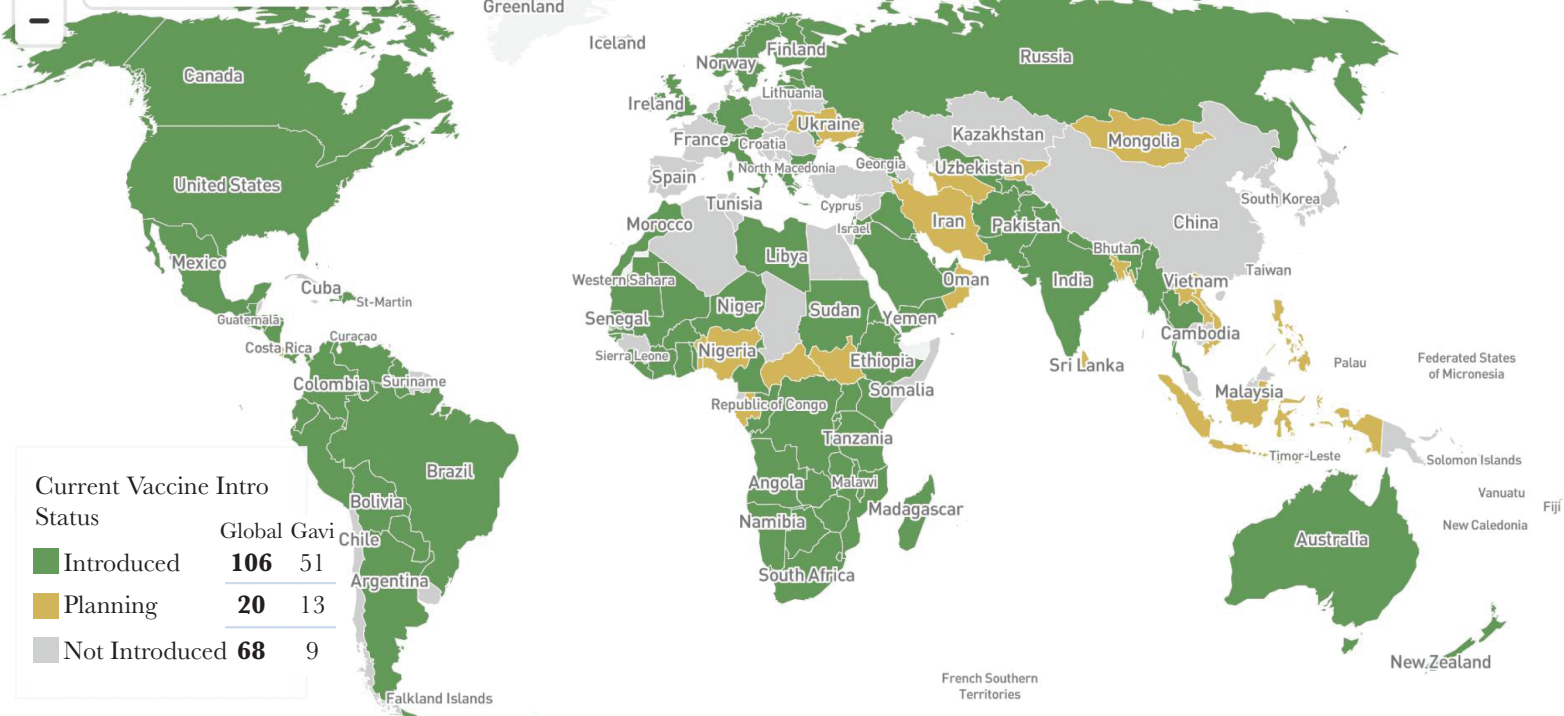
Countries that have introduced rotavirus vaccines into their national programs, January 2020 [54]. Colors indicate the countries being supported for introduction by the Global Alliance for Vaccines and Immunization (GAVI).

By 2020, over 100 countries had introduced rotavirus vaccines into their national routine immunization programs, a rapid uptake assisted both by the tiered pricing offered to middle-income countries by the manufacturers and subsidized funding from GAVI for low-income countries (ie, <$1026 per capita income) [54] (Figure 2). By region, vaccine uptake has been high in the Americas, Africa, and the Eastern Mediterranean region and lowest in Southeast Asia and Eastern Europe. Introduction in middle-income countries has lagged behind high- and low-income countries because despite its cost-effectiveness, in some settings, the price of this single vaccine exceeds the cost of most of the other childhood vaccines [55].

**Figure 3.**
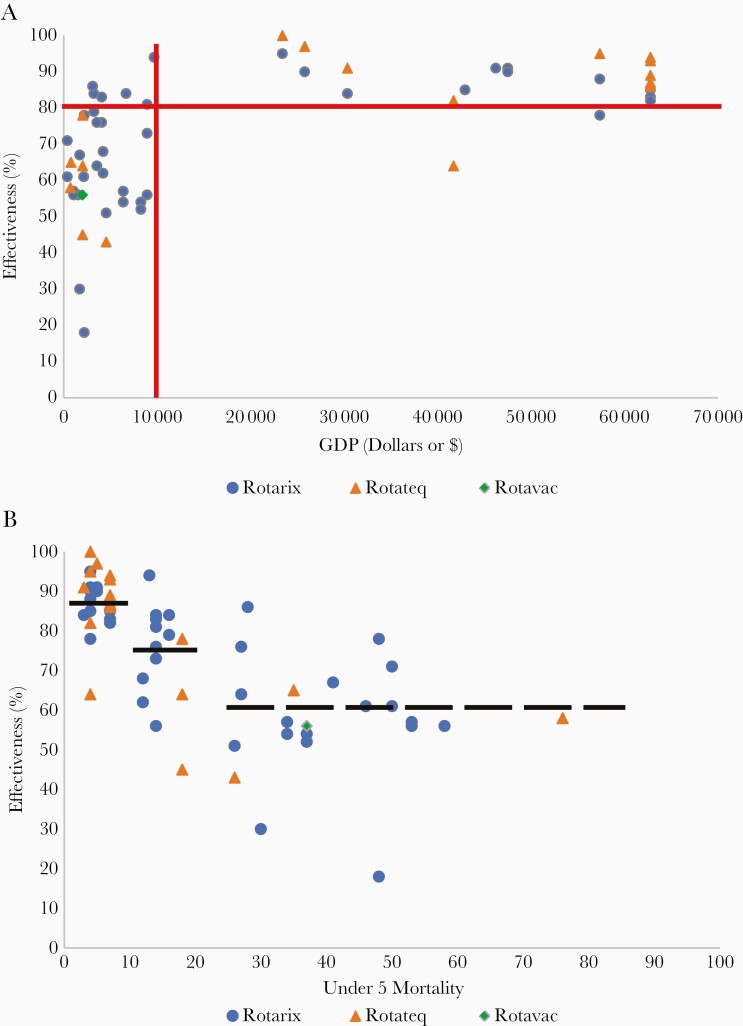
Effectiveness of oral rotavirus vaccines against severe rotavirus disease by country. *A*, Effectiveness versus per capita income. *B*, Effectiveness versus level of under 5 years mortality. Data by country and confidence intervals are available [58].

When the effectiveness of these live oral vaccines has been compared by countries based on their per capita income [56] and U5M, a troublesome trend was observed [57] (Figure 3A and 3B). Similar to earlier data on efficacy and immunogenicity from the clinical trials, a gradient was apparent. Effectiveness was greatest in countries with the lowest Under 5 mortality (Deaths/1000) (median 87%), intermediate (median 75%) in countries between 10 and 20 U5M, and lowest (median 60%) in countries with the highest U5M who could most benefit from the vaccine (Figure 3B). Vaccines were uniformly effective (median 89%) in all high-income settings (per capita > $12 375), but there was no difference in the median effectiveness (median range, 60%–62%) by income strata ranging from the countries with the lowest income (<$1026) to those in upper-middle income bracket ($3996–$12 375), although there was great variability in this range (Figure 3A).

Many studies have sought to determine why this is so. Malnutrition and stunting were significant factors [57, 59–61] but coinfection with other enteric pathogens did not appear to play a major role. Recent studies indicate that environmental enteropathy and alterations of the microbiome that impact nutrition can impact vaccine effectiveness [62–64]. Initially, age at vaccination seemed to be important with older children having a better immune response but this observation has not been consistently observed in postlicensure effectiveness studies [65–68]. Finally, the diversity of circulating rotavirus strains does not appear to be a major contributor [69, 70].

Despite a gradient in estimates of rotavirus vaccine effectiveness by setting, the impact of vaccine programs to decrease the burden of rotavirus disease has been substantial in countries across the mortality strata [71] (Figure 3B). Among countries that have introduced rotavirus vaccine into their routine immunization programs, rotavirus hospitalizations among children <5 years of age have been reduced by a median of 59%, acute gastroenteritis hospitalizations by 36%, and diarrhea mortality by 36% [71]. Reductions in hospitalizations and severe disease have been greatest in countries with low child mortality, among younger age groups where the rotavirus disease burden is greatest, and in countries with higher rotavirus vaccine coverage.

Some of the consequences from the introduction of rotavirus vaccines have been surprising. We have observed changes in the age distribution of rotavirus cases, changes in seasonal onset of disease, a marked reduction in the seasonal peak, and changes in the length of hospital stay. In hospitals, the proportion of diarrhea admissions for diarrhea due to rotavirus among children <5 years of age has decreased from approximately 40% in the prevaccine era to 20%–25% in the postvaccine introduction era, and these reductions have been sustained for 4 to 10 years postvaccine introduction [71, 72]. In some countries after rotavirus vaccine programs were introduced, indirect herd or community protection was observed early on as a reduction in rotavirus hospitalizations among older children who were not eligible to be vaccinated [71] and in adults [73, 74]. An unanticipated benefit of rotavirus vaccination has been a reduction in seizures following rotavirus vaccine introduction in several countries [75–79].

Although no increased risk of IS following rotavirus vaccination was observed in clinical trials, postlicensure monitoring was recommended. In several postlicensure evaluations of rotavirus vaccine in high- and middle-income countries, an increased risk of IS was observed in the 1–7 days after the first dose of rotavirus vaccine, resulting in 1 to 6 excess cases per 100 000 vaccinated infants [80, 81, 82, 83, 84, 85, 86]. However, no increased risk of IS has been seen following rotavirus vaccination in high-mortality countries in sub-Saharan Africa or in India [87, 88, 89, 90]. Reasons for this difference are unknown but may be due to several factors including lower immunogenicity of rotavirus vaccines in these settings, differences in age at administration, or interference by oral polio vaccine. No increased long-term risk of IS has been observed following rotavirus vaccine introduction [91].

WHO’s Global Advisory Committee on Vaccine Safety and the national immunization advisory committees in many countries have reviewed these data and all have reaffirmed their recommendation of rotavirus vaccine use stating the documented benefits of rotavirus vaccine far outweigh the small, short-term increased risk of IS following rotavirus vaccination [92].

## THE PIPELINE FOR NEW ROTAVIRUS VACCINES

Other candidate live oral vaccines are in development with the most advanced being RV3, a neonatal strain from Australia that is now in clinical trial (Table 1) [93]. An important and perhaps distinguishing feature of this vaccine has been its good immunogenicity when administered in the newborn period allowing for a birth dose. While rotavirus is less common in the first 2–3 months of life, infants do get rotavirus in this period and a birth dose could further decrease their ultimate risk of disease in their first 5 years of life.

Because live oral rotavirus vaccines are only approximately 60% protective in low-income countries with high U5M, and because vaccine coverage in many of these countries remains at approximately 70%, it has become clear that in these settings, live oral vaccines alone will not be able to completely control rotavirus disease [94]. This critical observation has led to new efforts to develop next-generation vaccines that might be delivered by injection or skin patches. If effective, these new vaccines could have several additional advantages [95]. For one, a concern for IS might not be shared with a parenteral or skin vaccine that is neither live nor oral. Parenteral vaccines could be combined with other injectable vaccines, such as IPV, or pentavalent or hexavalent vaccines, that would simplify administration, increase coverage, and alleviate administrative concerns for a separate supply chain or a greater volume of cold chain storage and decrease time in clinics. A combination 2 dose IPV—parenteral rotavirus vaccine could be used when oral polio vaccine is phased out. In the interim, as long as the IPV supply is limited, a single combination IRV-IPV might be used to boost the immune responses to both oral polio vaccine and oral rotavirus vaccines [96]. Finally, advances in the use of microneedles and skin immunization could provide an alternative, injection-free means of delivery, an approach that is now under investigation [97]. In short, research on next-generation vaccines will be essential if we hope to ultimately control the continuing burden of severe rotavirus disease in the future.

Several candidate parenteral rotavirus vaccines are currently in different stages of development [48] (Table 2). The most advanced is the nonreplicating rotavirus vaccine, (NRRV) patented by Hoshino at the National Institutes of Health and being developed by PATH and SK Biologics in Korea. The vaccine consists of a truncated VP8 subunit protein expressed in *Escherichia coli* and fused to the P2 epitope from tetanus toxin [98]. In trials in South African toddlers and infants, a monovalent formulation based upon the VP8 subunit from the P[8] rotavirus strain Wa generated robust neutralizing antibodies to the homologous P[8] strain but relatively modest responses to heterologous P[4] and P[6] strains [98]. Consequently, a trivalent vaccine formulation (P2-VP8-P[4],[6],[8]) was developed, which appears to have better neutralizing antibodies against each of the 3 vaccines P types [99]. In the initial phase 2 dosing studies, infants immunized intramuscularly with varying doses of NRRV were subsequently challenged with the live oral vaccine, Rotarix, to determine if their immunity would be sufficient to halt shedding of the live virus and whether the reduction in shedding could serve as an independent indicator of vaccine take. For Rotarix, shedding is common following the first dose but greatly reduced or eliminated after the second dose, a clear predictor of vaccine take. For NRRV, shedding following 2 doses of the subunit vaccine was only partially reduced and this reduction was not dose dependent. This may suggest either that additional antigens, presumably from other gene segments, may be required to improve the performance of this vaccine or that this parenteral vaccine does not provide the local immunity required to reduce shedding. A phase 3 trial is ongoing to determine the efficacy of this candidate as well as whether or not shedding could be a true correlate of protection.

**Table 2.** New Parenteral Rotavirus Vaccines in Development

Candidate	Principle	Developer	Status of Development	Comments
NRRV	VP8 fragments from 3 trivalent RV P-types P2-VP8-P[4],[6],[8] Expressed in *E. coli* Al(OH)_3_ adjuvant	PATH from NIH license SK Vaccines, Korea	Phase 2 trial completed neutralizing antibody to phase 3 trial ongoing (ClinicalTrials.gov NCT02646891)	Cross-neutralizing antibody responses Vaccinees challenged with Rotarix had partial decrease in shedding
IRV (CDC-9)	Inactivated RV strain CDC-9 for IM injection and skin immunization with a microneedle patch	Serum Institute of India and Chongqing Zhifei Biological Products, China from CDC license CDC/Micron Biomedical United States, microneedles	Proof of principle in animals Pilot lots in development IND pending (United States and China)	Intestinal immunity by both IM and microneedle patch immunization
IRV (ZTR-68))	Inactivated RV strain ZTR-68 for IM injection	Institute of Medical Biology, Kunming, China	Proof of principle in animals IND pending (China)	
P. 24-VP8* nanoparticle	Dual Norovirus P particle with expressing [P6],[P8] RV fragments of VP8 in nanoparticle	Cincinnati Children’s Hospital Medical Center	Proof of principle in piglets Pilot lot in development	
S60-VP8 pseudovirus nanoparticle	Norovirus nanoparticle carrying 4 VP8 fragments of [P8],[P4],[P6],[P11]	Cincinnati Children’s Hospital Medical Center	Early preclinical development	
VLP	VP2/6/7: VP2/4/6/7	Baylor College of Medicine	Proof of principle in small animals Process development	
mRNA vaccine	mRNA injected IM to induce expression of protein capsid in vaccinee	CureVac, Germany	Early development	
Plant-based VLP	VP2, VP6, VP7 from G1P[8] strain expressed In tobacco plants	Mitsubishi-Tanabe Pharma, Japan	Small animal studies given IM, IN, and oral Phase 1 study	
VP6-NV nanoparticle	Nanoparticle—self assembled norovirus capsid proteins with VP6 RV-baculovirus	University of Tampere, Finland	Strong humoral and T-cell immunity Protection in small animal studies against RV	

Abbreviations: CDC, Centers for Disease Control and Prevention; IM, intramuscular; IND, Investigational New Drug Application; IRV, inactivated rotavirus vaccine; NIH, National Institutes of Health; NRRV, Nonreplicating rotavirus vaccine; RV, rotavirus; VLP, virus-like particle.

Another nonreplicating rotavirus candidate initially developed at the US CDC is a fully inactivated rotavirus vaccine (IRV) strain (CDC-9) that has demonstrated proof of principle in the gnotobiotic piglet model [100]. Unlike NRRV, IRV has the full complement of genes from a most common rotavirus strain, G1P[8]. This unique human strain grows to high titer in Vero cells and remains relatively intact throughout the purification process. Tests in the gnotobiotic piglet model demonstrated the intramuscular immunization gave broad neutralizing activity and significantly reduced shedding and diarrhea from a live rotavirus challenge [101]. IRV has also been formulated to be administered by skin immunization using a microneedle patch. The patch induced an immune response comparable to the intramuscular injection with substantial sparing of antigen in animal studies [97]. Phase 1 trials for intramuscular and intradermal immunizations are due to begin in 2021. Another IRV strain (ZTR-68) is also under development in China [102].

Virus-like particles (VLPs) with multiple gene segments have been prepared as candidates by 2 groups [48]. In Japan, researchers at Mitsubishi Tanabe Pharma have incorporated genes encoding 3 gene segments, VP2, VP6, and VP7, into tobacco (*Nicotiana benthamiana*) using *Agrobacterium* vectors transfected with the rotavirus genes and have purified the VLPs into a parenteral vaccine. Phase 1 studies have demonstrated both safety and immunogenicity. At Baylor, different combinations of gene segments (VP2/6/7 and VP2/4/6/7) have been expressed in baculovirus to produce VLPs. These candidates when administered by both the intramuscular and intranasal route induced a good immune response and protected animals from homotypic challenge [103].

Two vaccine candidates have been developed from single-gene segments from rotavirus expressed in nanoparticles derived from subunits of norovirus protein [48]. In Cincinnati, P24-VP8 nanoparticles expressing VP8 fragments of P[6] and P[8] serotypes have demonstrated good immune response and proof of principle in piglet studies and are in further development [104]. In Finland, a nanoparticle construct derived from self-assembled norovirus capsid proteins has been engineered to carry a VP6 structural gene. This candidate induced both humoral and T-cell immunity and protection in small animal studies [105]. Both of these candidates are intended to provide dual protection against both rotavirus and norovirus but proof of principle against norovirus has not yet been tested. Neither has been in phase 1 trials in humans.

## THE FUTURE CONTROL OF ROTAVIRUS DIARRHEA

**Figure 4.**
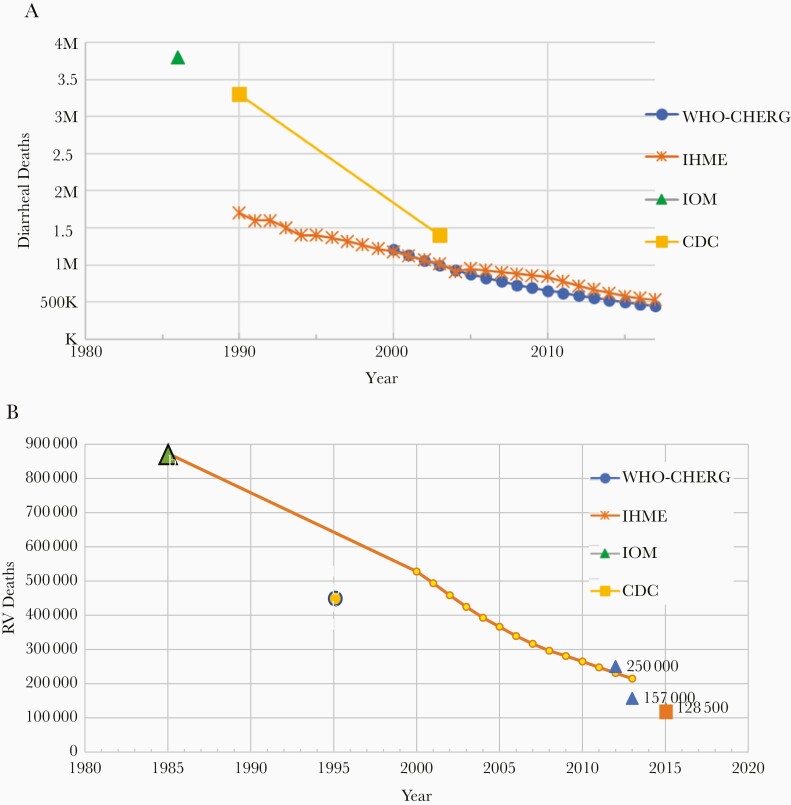
Estimated annual numbers of diarrheal and rotavirus deaths in children <5 years, from 1980 to 2017. *A*, Total diarrheal deaths [14, 106, 108, 109, 111]. *B*, Rotavirus deaths [14, 107, 110, 111]. Abbreviations: CDC, Centers for Disease Control and Prevention; IHME, Institute for Health Metrics and Evaluation; IOM, Institute of Medicine; K, thousand; M, million; RV, rotavirus; WHO-CHERG, World Health Organization Child Health Epidemiology Reference Group.

In the nearly 5 decades since the discovery of rotavirus, the estimated childhood mortality from diarrhea has decreased markedly, from approximately 3.6 million deaths in 1986 to approximately 500 000 in 2018 (Figure 4). Much of this improvement came in the approximately 35 years before rotavirus vaccines were introduced. Mathematical models have estimated that these declines in mortality in the poorest countries can be attributed to improved treatment with rehydration therapy, breastfeeding, birth-spacing, education and delayed pregnancies of the mothers, smaller family sizes, and improvements in water, sanitation, and hygiene [106]. Since 2009, the WHO has recommended that rotavirus vaccine be used in all countries and GAVI has subsidized vaccine purchase by low-income countries. Since 2018, the ramping up of production by the 2 new Indian manufacturers has made rotavirus vaccines available today to an estimated 54% of the world’s children in approximately 100 countries. The world has now witnessed a major decrease in the global burden of severe rotavirus disease. By 2018, estimates of the annual number of deaths from rotavirus have been reduced to approximately 150 000 to 200 000 [107] and the percent of severe diarrhea attributed to diarrheal hospitalizations has fallen from approximately 34%–40% to 20%–24% with a wide range by country.

What can be done to further reduce the burden of rotavirus diarrhea going forward? To fully control rotavirus disease worldwide, several strategies will be required. First, rotavirus vaccine needs to be implemented in the approximately 90 countries hosting approximately 46% of the world’s children where rotavirus immunization has not yet been introduced into the national immunization programs. This will require that policy makers reassess the local burden of disease and determine the price point at which implementing a national program would be worthwhile and for manufacturers to lower vaccine cost over time as volume expands.

At the same time, a robust research agenda is underway to develop the next generation of parenteral or skin patch rotavirus vaccines that might be more effective and ultimately replace the oral vaccines. If these vaccines prove to be safe, more effective, easier to administer in a combined vaccine formulation, and affordable to all, the eventual control of the disease could be achieved.

The vision to control rotavirus diarrhea as a means to improve the health and survival of all children has come a long way since the discovery of the virus in 1973, from a global aspiration to today when 4 globally licensed vaccines are being used routinely in more than 100 countries. At the same time, while low-income countries that have introduced rotavirus into their national immunization programs have benefitted greatly from this intervention, they remain today with rotavirus still the first or second most common cause of diarrheal hospitalizations and diarrheal deaths. A continued effort to develop more effective vaccines will be essential to ultimately control this disease on a worldwide basis, especially in those countries with high mortality from diarrhea that will need these vaccines the most.

## Supplementary Data

Supplementary materials are available at *The Journal of Infectious Diseases* online. Consisting of data provided by the authors to benefit the reader, the posted materials are not copyedited and are the sole responsibility of the authors, so questions or comments should be addressed to the corresponding author.

The complete references are available as online Supplemental Material.

## Supplementary Material

jiaa598_suppl_Supplementary-Material

## Notes

***Acknowledgments.*** The authors acknowledge the help of D. Carl Kirkwood, A. Duncan Steele, Mary K. Estes, Jason Jiang, and Michele Groome for their helpful comments, John Patton for providing Figure 1, and Kristen Weymouth and Ann Puderbaugh for helpful edits and artwork.

***Supplement sponsorship.*** This supplement is sponsored by the Bill and Melinda Gates Foundation.

***Potential******conflicts******of interest.*** B. J. and R. I. G. are inventors on government patents related to the inactivated rotavirus vaccine and R. I. G. was involved in the discovery and development of Rotavac but have no financial interest in these products. All authors: No reported conflicts of interest. All authors have submitted the ICMJE Form for Disclosure of Potential Conflicts of Interest. Conflicts that the editors consider relevant to the content of the manuscript have been disclosed.
