# Supplementary material for: The Rotavirus Vaccine Story: From Discovery to the Eventual Control of Rotavirus Disease
Source: J Infect Dis. 2021 Sep 30;224(Suppl 4):S331–42. doi: 10.1093/infdis/jiaa598 (PMC8482027; doi:10.1093/infdis/jiaa598)
Supplement: jiaa598_suppl_Supplementary-Material [file jiaa598_suppl_supplementary-material.docx]

Supplemental References:

1. Roser M. Our world in data. https://ourworldindata.org/life-expectancy-globally. Accessed 24 May 2020.

2. GBD 2017 Mortality Collaborators GBDM. Global, regional, and national age-sex-specific mortality and life expectancy, 1950-2017: a systematic analysis for the Global Burden of Disease Study 2017. Lancet 2018; 392:1684–735.

3. Snyder JD, Merson MH. The magnitude of the globalproblem of acute diarrhoeal disease: a review of active surveillance data. Bull World Health Organ 1982; 60:605–13.

4. Puffer RC, Serrano CV. Patterns of mortality in childhood.PAHO Scientific Publication No 262. Washington, DC: Pan American Health Organization, 1973.

5. Mata L. The Children of Santa Maria Cauqu 6. A Prospective Field Study of Health and Growth. Cambridge, MA: MIT Press, 1978:499.

6. Guerrant RL, Kirchhoff LV, Shields DS, et al. Prospective study of diarrheal illnesses in northeastern Brazil: patterns of disease, nutritional impact, etiologies, and risk factors. J Infect Dis 1983; 148:986–97.

7. Rohde JE, Northrup RS. Taking science where the diarrhoea is. Ciba Found Symp 1976; 42:339–58.

8. Black RE, Brown KH, Becker S, Alim AR, Huq I. Longitudinal studies of infectious diseases and physical growth of children in rural Bangladesh. II. Incidence of diarrhea and association with known pathogens. Am J Epidemiol 1982; 115:315–24.

9. Lanata CF, Black RE, Gilman RH, Lazo F, Del Aguila R. Epidemiologic, clinical, and laboratory characteristics of acute vs. persistent diarrhea in periurban Lima, Peru. J

Pediatr Gastroenterol Nutr 1991; 12:82–8.

10. Kapikian AZ, Wyatt RG, Dolin R, Thornhill TS, Kalica AR,

Chanock RM. Visualization by immune electron microscopy of a 27-nm particle associated with acute infectious nonbacterial gastroenteritis. J Virol 1972; 10:1075–81.

11. Bishop RF, Davidson GP, Holmes IH, Ruck BJ. Virus particles in epithelial cells of duodenal mucosa from children with acute non-bacterial gastroenteritis. Lancet 1973; 2:1281–3.

12. World Health Organization. WHO global invasive bacterial vaccine-preventable disease and rotavirus and pediatric surveillance networks bulletins. https://www.who.int/immunization/monitoring_surveillance/resources/NUVI/en/. Accessed 24 May 2020.

13. Velázquez FR, Matson DO, Calva JJ, et al. Rotavirus infection in infants as protection against subsequent infections.N Engl J Med 1996; 335:1022–8.

14. Institute of Medicine. The prospects of immunizing against rotavirus. New vaccine development: diseases of importance in developing countries. Washington, DC: National Academy Press, 1986.

15. Estes MK, Kapikian AZ. Rotaviruses. In: Knipe DM, Howley PM, eds. Field’s virology. 5th ed. Philadelphia, PA:Lippincott, Williams, and Williams, 2007:1917–58.

16. Patton JT. Rotavirus diversity and evolution in the postvaccine world. Discov Med 2012; 13:85–97.

17. Gentsch JR, Glass RI, Woods P, et al. Identification of group A rotavirus gene 4 types by polymerase chain reaction. J Clin Microbiol 1992; 30:1365–73.

18. Midthun K, Greenberg HB, Hoshino Y, Kapikian AZ, Wyatt RG, Chanock RM. Reassortant rotaviruses as potential live rotavirus vaccine candidates. J Virol 1985;53:949–54.

19. Matthijnssens J, Ciarlet M, McDonald SM, et al. Uniformity of rotavirus strain nomenclature proposed by the RotavirusClassification Working Group (RCWG). Arch Virol 2011;156:1397–413.

20. Estes MK, Cohen J. Rotavirus gene structure and function.Microbiol Rev 1989; 53:410–49.

21. Vesikari T, Isolauri E, D’Hondt E, Delem A, André FE,Zissis G. Protection of infants against rotavirus diarrhoea by RIT 4237 attenuated bovine rotavirus strain vaccine.Lancet 1984; 1:977–81.

22. Clark HF, Offit PA, Glass RI, Ward RL. Rotavirus vaccines. In: Plotkin SA, Orenstein WA, Offit PA, eds. Vaccines. 4^th^ ed. Philadelphia, PA: Elsevier, 2001:1327–45.

23. Kapikian AZ, Hoshino Y, Chanock RM, Pérez-Schael I. Efficacy of a quadrivalent rhesus rotavirus-based human rotavirus vaccine aimed at preventing severe rotavirus diarrhea in infants and young children. J Infect Dis 1996;174(Suppl 1):S65–72.

24. Pérez-Schael I, Guntiñas MJ, Pérez M, et al. Efficacy of the rhesus rotavirus-based quadrivalent vaccine in infants and young children in Venezuela. N Engl J Med 1997; 337:1181–7.

25. Bernstein DI, Glass RI, Rodgers G, Davidson BL, Sack DA. Evaluation of rhesus rotavirus monovalent and tetravalent reassortant vaccines in US children. US Rotavirus Vaccine Efficacy Group. JAMA 1995; 273:1191–6.

26. Rennels MB, Glass RI, Dennehy PH, et al. Safety and efficacy of high-dose rhesus-human reassortant rotavirus vaccines–report of the National Multicenter Trial. United States Rotavirus Vaccine Efficacy Group. Pediatrics 1996; 97:7–13.

27. Lanata CF, Midthun K, Black RE, et al. Safety, immunogenicity, and protective efficacy of one and three doses of the tetravalent rhesus rotavirus vaccine in infants in Lima, Peru. J Infect Dis 1996; 174:268–75.

28. Linhares AC, Gabbay YB, Mascarenhas JD, et al. Immunogenicity, safety and efficacy of tetravalent rhesushuman, reassortant rotavirus vaccine in Belém, Brazil. Bull World Health Organ 1996; 74:491–500.

29. Rotavirus vaccine for the prevention of rotavirus gastroenteritis among children. Recommendations of the Advisory Committee on Immunization Practices (ACIP). MMWR Recomm Rep 1999; 48:1–20.

30. Centers for Disease Control and Prevention. Intussusception among recipients of rotavirus vaccine--United States, 1998–1999. MMWR Morb Mortal Wkly Rep 1999; 48:577–81.

31. Murphy TV, Gargiullo PM, Massoudi MS, et al; Rotavirus Intussusception Investigation Team. Intussusception among infants given an oral rotavirus vaccine. N Engl J Med 2001; 344:564–72.

32. Centers for Disease Control and Prevention. Withdrawal of rotavirus vaccine recommendation. MMWR Morb Mortal Wkly Rep 1999; 48:1007.

33. Robinson CG, Hernanz-Schulman M, Zhu Y, Griffin MR, Gruber W, Edwards KM. Evaluation of anatomic changes in young children with natural rotavirus infection: is intussusception biologically plausible? J Infect Dis 2004; 189:1382–7.

34. Patel MM, Pitzer VE, Alonso WJ, et al. Global seasonality of rotavirus disease. Pediatr Infect Dis J 2013; 32:e134–47.

35. Ruiz-Palacios GM, Pérez-Schael I, Velázquez FR, et al; Human Rotavirus Vaccine Study Group. Safety and efficacy of an attenuated vaccine against severe rotavirus gastroenteritis. N Engl J Med 2006; 354:11–22.

36. Vesikari T, Matson DO, Dennehy P, et al; Rotavirus Efficacy and Safety Trial (REST) Study Team. Safety and efficacy of a pentavalent human-bovine (WC3) reassortant rotavirus vaccine. N Engl J Med 2006; 354:23–33.

37. Conclusions and recommendations from the Immunization Strategic Advisory Group. Wkly Epidemiol Rec 2006;81:2–11.

38. Zaman K, Dang DA, Victor JC, et al. Efficacy of pentavalent rotavirus vaccine against severe rotavirus gastroenteritis in infants in developing countries in Asia: a randomised, double-blind, placebo-controlled trial. Lancet 2010; 376:615–23.

39. Armah GE, Sow SO, Breiman RF, et al. Efficacy of pentavalent rotavirus vaccine against severe rotavirus gastroenteritis in infants in developing countries in sub-Saharan Africa: a randomised, double-blind, placebo-controlled trial. Lancet 2010; 376:606–14.

40. Madhi SA, Cunliffe NA, Steele D, et al. Effect of human rotavirus vaccine on severe diarrhea in African infants. N Engl J Med 2010; 362:289–98.

41. Patel M, Shane AL, Parashar UD, Jiang B, Gentsch JR, Glass RI. Oral rotavirus vaccines: how well will they work where they are needed most? J Infect Dis 2009; 200(Suppl 1):S39–48.

42. Meeting of the Immunization Strategic Advisory Group of Experts, April 2009--conclusions and recommendations. Wkly Epidemiol Rec 2009; 84:220–36.

43. Soares-Weiser K, Bergman H, Henschke N, Pitan F, Cunliffe N. Vaccines for preventing rotavirus diarrhoea: vaccines in use. Cochrane Database Syst Rev 2019; (10):CD008521.

44. Bhandari N, Rongsen-Chandola T, Bavdekar A, et al; India

Rotavirus Vaccine Group. Efficacy of a monovalent humanbovine (116E) rotavirus vaccine in Indian infants: a randomised, double-blind, placebo-controlled trial. Lancet 2014; 383:2136–43.

45. Kulkarni PS, Desai S, Tewari T, et al; SII BRV-PV author group. A randomized Phase III clinical trial to assess the efficacy of a bovine-human reassortant pentavalent rotavirus vaccine in Indian infants. Vaccine 2017; 35:6228–37.

46. Isanaka S, Guindo O, Langendorf C, et al. Efficacy of a lowcost, heat-stable oral rotavirus vaccine in Niger. N Engl J Med 2017; 376:1121–30.

47. Bhan MK, Glass RI, Ella KM, et al. Team science and the creation of a novel rotavirus vaccine in India: a new framework for vaccine development. Lancet 2014; 383:2180–3.

48. Kirkwood CD, Ma LF, Carey ME, Steele AD. The rotavirus vaccine development pipeline. Vaccine 2019; 37:7328–35.

49. Dang DA, Nguyen VT, Vu DT, et al; Rotavin-M1 Vaccine Trial Group. A dose-escalation safety and immunogenicity study of a new live attenuated human rotavirus vaccine (Rotavin-M1) in Vietnamese children. Vaccine 2012; 30(Suppl 1):A114–21.

50. Kirkwood CD, Steele AD. Rotavirus vaccines in China:improvement still required. JAMA Netw Open 2018; 1:e181579.

51. Fu C, He Q, Xu J, et al. Effectiveness of the Lanzhou lamb rotavirus vaccine against gastroenteritis among children. Vaccine 2012; 31:154–8.

52. Fu C, Tate JE, Jiang B. Effectiveness of Lanzhou lamb rotavirus vaccine against hospitalized gastroenteritis: further analysis and update. Hum Vaccin 2010; 6:953.

53. Zhen SS, Li Y, Wang SM, et al. Effectiveness of the live attenuated rotavirus vaccine produced by a domestic manufacturer in China studied using a population-based case-control design. Emerg Microbes Infect 2015; 4:e64.

54. International Vaccine Access Center, Johns Hopkins Bloomberg School of Public Health. Current vaccine intro status. https://view-hub.org/map/?set=current-vaccineintro-status&category=rv&group=vaccine-introduction.Accessed 25 October 2020.

55. Debellut F, Clark A, Pecenka C, et al. Re-evaluating the potential impact and cost-effectiveness of rotavirus vaccination in 73 Gavi countries: a modelling study. Lancet Glob Health 2019; 7:e1664–74.

56. World Bank. Open data https://data.worldbank.org/.Accessed 22 August 2020.

57. Jonesteller CL, Burnett E, Yen C, Tate JE, Parashar UD. Effectiveness of rotavirus vaccination: a systematic review of the first decade of global postlicensure data, 2006–2016. Clin Infect Dis 2017; 65:840–50.

58. Burnett E, Parashar UD, Tate JE. Real-world effectiveness of rotavirus vaccines, 2006-19: a literature review and metaanalysis. Lancet Glob Health 2020; 8:e1195–202.

59. Mujuru HA, Burnett E, Nathoo KJ, et al. Monovalent rotavirus vaccine effectiveness against rotavirus hospitalizations among children in Zimbabwe. Clin Infect Dis 2019;69:1339–44.

60. Bar-Zeev N, Jere KC, Bennett A, et al; Vaccine Effectiveness and Disease Surveillance Programme, Malawi (VACSURV) Consortium. Population impact and effectiveness of monovalent rotavirus vaccination in urban Malawian children 3 years after vaccine introduction: ecological and casecontrol analyses. Clin Infect Dis 2016; 62(Suppl 2):S213–9.

61. Khagayi S, Omore R, Otieno GP, et al. Effectiveness of monovalent rotavirus vaccine against hospitalization with acute rotavirus gastroenteritis in Kenyan children. Clin Infect Dis

2020; 70:2298–305.

62. Mokomane M, Tate JE, Steenhoff AP, et al. Evaluation of the influence of gastrointestinal coinfections on rotavirus vaccine effectiveness in Botswana. Pediatr Infect Dis J 2018;37:e58–62.

63. Groome MJ, Page N, Cortese MM, et al. Effectiveness of monovalent human rotavirus vaccine against admission to hospital for acute rotavirus diarrhoea in South African children: a case-control study. Lancet Infect Dis 2014;14:1096–104.

64. Chen RY, Kung VL, Das S, et.al. Duodenal microbiota in stunted undernourished children with enteropathy. New Engl J Med 2020; 383:321–33.

65. Steele AD, De Vos B, Tumbo J, et al. Co-administration study in South African infants of a live-attenuated oral human rotavirus vaccine (RIX4414) and poliovirus vaccines. Vaccine 2010; 28:6542–8.

66. Anh DD, Carlos CC, Thiem DV, et al. Immunogenicity, reactogenicity and safety of the human rotavirus vaccine RIX4414 (Rotarix) oral suspension (liquid formulation) when co-administered with expanded program on immunization (EPI) vaccines in Vietnam and the Philippines in 2006–2007. Vaccine 2011; 29:2029–36.

67. Armah G, Lewis KD, Cortese MM, et al. A randomized, controlled trial of the impact of alternative dosing schedules on the immune response to human rotavirus vaccine in rural Ghanaian infants. J Infect Dis 2016; 213:1678–85.

68. Burke RM, Tate JE, Pringle KD, Patel M, De Oliveira LH, Parashar UD. Effect of age at vaccination on rotavirus vaccine effectiveness in Bolivian infants. Pediatr Infect Dis J 2018; 37:e216–21.

69. Leshem E, Lopman B, Glass R, et al. Distribution of rotavirus strains and strain-specific effectiveness of the rotavirus vaccine after its introduction: a systematic review and meta-analysis. Lancet Infect Dis 2014; 14:847–56.

70. Velasquez DE, Parashar UD, Jiang B. Strain diversity plays no major role in the varying efficacy of rotavirus vaccines: an overview. Infect Genet Evol 2014; 28:561–71.

71. Burnett E, Parashar UD, Tate JE. Global impact of rotavirus vaccination on diarrhea hospitalizations and deaths among children <5 years old: 2006–2019 [published online ahead of print 25 February 2020]. J Infect Dis doi: 10.1093/infdis/ jiaa081.

72. Aliabadi N, Antoni S, Mwenda JM, et al. Global impact of rotavirus vaccine introduction on rotavirus hospitalisations among children under 5 years of age, 2008-16: findings from the Global Rotavirus Surveillance Network. Lancet Glob Health 2019; 7:e893–903.

73. Lopman BA, Curns AT, Yen C, Parashar UD. Infant rotavirus vaccination may provide indirect protection to older children and adults in the United States. J Infect Dis 2011; 204:980–6.

74. Gastañaduy PA, Curns AT, Parashar UD, Lopman BA. Gastroenteritis hospitalizations in older children and adults in the United States before and after implementation of infant rotavirus vaccination. JAMA 2013; 310:851–3.

75. Payne DC, Baggs J, Zerr DM, et al. Protective association between rotavirus vaccination and childhood seizures in the year following vaccination in US children. Clin Infect Dis 2014; 58:173–7.

76. Sheridan SL, Ware RS, Grimwood K, Lambert SB. Febrile Seizures in the Era of Rotavirus Vaccine. J Pediatric Infect Dis Soc 2016; 5:206–9.

77. Pardo-Seco J, Cebey-López M, Martinón-Torres N, et al. Impact of rotavirus vaccination on childhood hospitalization for seizures. Pediatr Infect Dis J 2015; 34:769–73.

78. Pringle KD, Burke RM, Steiner CA, Parashar UD, Tate JE. Trends in rate of seizure-associated hospitalizations among children <5 years old before and after rotavirus vaccine introduction in the United Sates, 2000–2013. J Infect Dis 2018; 217:581–8.

79. Burke RM, Tate JE, Dahl RM, Aliabadi N, Parashar UD. Rotavirus vaccination is associated with reduced seizure hospitalization risk among commercially insured US children. Clin Infect Dis 2018; 67:1614–6.

80. Patel MM, López-Collada VR, Bulhões MM, et al. 81. Carlin JB, Macartney KK, Lee KJ, et al. Intussusception risk and disease prevention associated with rotavirus vaccines in Australia’s national immunization program. Clin Infect Dis 2013; 57:1427–34.

82. Weintraub ES, Baggs J, Duffy J, et al. Risk of intussusception after monovalent rotavirus vaccination. N Engl J Med 2014; 370:513–9.

83. Stowe J, Andrews N, Ladhani S, Miller E. The risk of intussusception following monovalent rotavirus vaccination in England: a self-controlled case-series evaluation. Vaccine 2016; 34:3684–9.

84. Velázquez FR, Colindres RE, Grajales C, et al. Postmarketing surveillance of intussusception following mass introduction of the attenuated human rotavirus vaccine in Mexico. Pediatr Infect Dis J 2012; 31:736–44.

85. Yih WK, Lieu TA, Kulldorff M, et al. Intussusception risk after rotavirus vaccination in U.S. infants. N Engl J Med 2014; 370:503–12.

86. Yung CF, Chan SP, Soh S, Tan A, Thoon KC. Intussusception and monovalent rotavirus vaccination in Singapore:self-controlled case series and risk-benefit study. J Pediatr 2015; 167:163–8.e1.

87. Tate JE, Mwenda JM, Armah G, et al; African Intussusception Surveillance Network. Evaluation of intussusception after monovalent rotavirus vaccination in Africa. N Engl J Med 2018; 378:1521–8.

88. Groome MJ, Tate JE, Arnold M, et al. Evaluation of intussusception after oral monovalent rotavirus vaccination in South Africa. Clin Infect Dis 2020; 70:1606–12. 89. World Health Organization. Global Advisory Committee on Vaccine Safety,4–5 December 2019. <http://www.who>. int/vaccine_safety/committee/reports/Dec_2019/en/.Accessed 15 March 2020.

90. Reddy S, Nair NP, Tate JE, et al. Intussusception after rotavirus vaccine introduction in India. N Engl J Med 2020; 383:1932-1940.

91. Burke RM, Tate JE, Dahl RM, Aliabadi N, Parashar UD. Does rotavirus vaccination affect longer-term intussusception risk in US infants? J Pediatric Infect Dis Soc 2020; 9:257–60.

92. Rotavirus vaccines. WHO position paper—January 2013. Wkly Epidemiol Rec 2013; 88:49–64.

93. Bines JE, At Thobari J, Satria CD, et al. Human neonatal rotavirus vaccine (RV3-BB) to target rotavirus from birth. N Engl J Med 2018; 378:719–30.

94. Glass RI, Jiang B, Parashar U. The future control of rotavirus disease: can live oral vaccines alone solve the rotavirus problem? Vaccine 2018; 36:2233–6.

95. Jiang B, Gentsch JR, Glass RI. Inactivated rotavirus vaccines: a priority for accelerated vaccine development. Vaccine 2008; 26:6754–8.

96. Jiang B, Patel M, Glass RI. Polio endgame: lessons for the global rotavirus vaccination program. Vaccine 2019; 37:3040–9.

97. Resch TK, Wang Y, Moon SS, et al. Inactivated rotavirus vaccine by parenteral administration induces mucosal immunity in mice. Sci Rep 2018; 8:561.

98. Groome MJ, Koen A, Fix A, et al. Safety and immunogenicity of a parenteral P2-VP8-P[8] subunit rotavirus vaccine in toddlers and infants in South Africa: a randomised, double-blind, placebo-controlled trial. Lancet Infect Dis 2017; 17:843–53.

99. Groome MJ, Fairlie L, Morrison J, et al. Safety and immunogenicity of a parenteral trivalent P2-VP8 subunit rotavirus vaccine: a multisite, randomised, double-blind, placebo-controlled trial. Lancet Infect Dis 2020; 20:851–63.

100. Wang Y, Vlasova A, Velasquez DE, et al. Skin vaccination against rotavirus using microneedles: proof of concept in gnotobiotic piglets. PLoS One 2016; 11:e0166038.

101. Jiang B, Wang Y, Glass RI. Does a monovalent inactivated human rotavirus vaccine induce heterotypic immunity? Evidence from animal studies. Hum Vaccin Immunother 2013; 9:1634–7.

102. Wu JY, Zhou Y, Zhang GM, et al. Isolation and characterization of a new candidate human inactivated rotavirus vaccine strain from hospitalized children in Yunnan, China: 2010–2013. World J Clin Cases 2018; 6:426–40.

103. Ciarlet M, Crawford SE, Barone C, et al. Subunit rotavirus vaccine administered parenterally to rabbits induces active protective immunity. J Virol 1998; 72:9233–46.

104. Ramesh A, Mao J, Lei S, et al. Parenterally administered P24-VP8* nanoparticle vaccine conferred strong protection against rotavirus diarrhea and virus shedding in gnotobiotic pigs. Vaccines (Basel) 2019; 7:177.

105. Blazevic V, Malm M, Arinobu D, Lappalainen S, Vesikari T.Rotavirus capsid VP6 protein acts as an adjuvant in vivo for norovirus virus-like particles in a combination vaccine. Hum Vaccin Immunother 2016; 12:740–8.

106. Black R, Fontaine O, Lamberti L, et al. Drivers of the reduction in childhood diarrhea mortality 1980–2015 and interventions to eliminate preventable diarrhea deaths by 2030. J Glob Health 2019; 9:020801.

107. Troeger C, Khalil IA, Rao PC, et al. Rotavirus vaccination and the global burden of rotavirus diarrhea among children younger than 5 years. JAMA Pediatr 2018; 172:958–65.

108. Bern C, Martines J, de Zoysa I, Glass RI. The magnitude

of the global problem of diarrhoeal disease: a ten-year update. Bull World Health Organ 1992; 70:705–14.

109. Parashar UD, Hummelman EG, Bresee JS, Miller MA, Glass RI. Global illness and deaths caused by rotavirus disease in children. Emerg Infect Dis 2003; 9:565–72.

110. Clark A, Black R, Tate J, et al. Estimating global, regional and national rotavirus deaths in children aged <5 years: current approaches, new analyses and proposed improvements. PLoS One 2017;12:e0183392.

111. Dadonaite B, Ritchie H, Roser, M. Diarrheal diseases, 2019. https://ourworldindata.org/diarrheal-diseases. Accessed 25 August 2020.
